# Rationale, design, methodology and sample characteristics for the family partners for health study: a cluster randomized controlled study

**DOI:** 10.1186/1471-2458-12-250

**Published:** 2012-03-30

**Authors:** Diane C Berry, Robert McMurray, Todd A Schwartz, Anne Skelly, Maria Sanchez, Madeline Neal, Gail Hall

**Affiliations:** 1 School of Nursing, The University of North Carolina at Chapel Hill, Campus Box 7460, Chapel Hill, NC 27599-7460, USA; 2 School of Exercise and Sport Science, The University of North Carolina at Chapel Hill, 025 Fetzer Gymnasium, Campus Box 8700, Chapel Hill, NC 27599-8700, USA

## Abstract

**Background:**

Young children who are overweight are at increased risk of becoming obese and developing type 2 diabetes and cardiovascular disease later in life. Therefore, early intervention is critical. This paper describes the rationale, design, methodology, and sample characteristics of a 5-year cluster randomized controlled trial being conducted in eight elementary schools in rural North Carolina, United States.

**Methods/Design:**

The first aim of the trial is to examine the effects of a two-phased intervention on weight status, adiposity, nutrition and exercise health behaviors, and self-efficacy in overweight or obese 2nd, 3 rd, and 4th grade children and their overweight or obese parents. The primary outcome in children is stabilization of BMI percentile trajectory from baseline to 18 months. The primary outcome in parents is a decrease in BMI from baseline to 18 months. Secondary outcomes for both children and parents include adiposity, nutrition and exercise health behaviors, and self-efficacy from baseline to 18 months. A secondary aim of the trial is to examine in the experimental group, the relationships between parents and children's changes in weight status, adiposity, nutrition and exercise health behaviors, and self-efficacy. An exploratory aim is to determine whether African American, Hispanic, and non-Hispanic white children and parents in the experimental group benefit differently from the intervention in weight status, adiposity, health behaviors, and self-efficacy.

A total of 358 African American, non-Hispanic white, and bilingual Hispanic children with a BMI ≥ 85th percentile and 358 parents with a BMI ≥ 25 kg/m^2 ^have been inducted over 3 1/2 years and randomized by cohort to either an experimental or a wait-listed control group. The experimental group receives a 12-week intensive intervention of nutrition and exercise education, coping skills training and exercise (Phase I), 9 months of continued monthly contact (Phase II) and then 6 months (follow-up) on their own. Safety endpoints include adverse event reporting. Intention-to-treat analysis will be applied to all data.

**Discussion:**

Findings from this trial may lead to an effective intervention to assist children and parents to work together to improve nutrition and exercise patterns by making small lifestyle pattern changes.

**Trial registration:**

NCT01378806.

## Background

Overweight and obesity in children and adults is a global health concern [1]. In the United States (U.S.), 68% of adults are either overweight (body mass index [BMI] ≥ 25 kg/m^2^) or obese (BMI ≥ 30 kg/m^2^) [2]; 38% of children are either overweight (≥ 85^th ^%) or obese (≥ 95^th ^%) [3,4]. Overweight is prevalent in both genders, affects all ages, and crosses all ethnic groups, though ethnic minorities and individuals with lower incomes and less education are most affected [1,3,4].

Obesity contributes to many preventable chronic diseases, including hypertension, hypercholesterolemia, cardiovascular disease, sleep apnea, degenerative joint disease, depression, prediabetes and type 2 diabetes [5,6]. Overweight children are more likely to become overweight or obese adults [5,6], and obese adults have more medical expenditures, are more likely to be hospitalized, have longer lengths of stay, and have higher inpatient, outpatient, and pharmacy costs [7]. Total annual medical expenditures related to obesity now exceed $300 billion in direct and indirect costs per year in the U.S [8]. As a result of the adverse outcomes associated with overweight and obesity, for the first time in our history a generation of children in the U.S. is projected to have a lower life expectancy than their parents [9].

The quality of nutritional intake in children and adults in the U.S. has declined over the past two decades as intake of calorie-dense and high fat foods has increased [10,11]. Children drink less milk and more juice and soda [12], and fewer than 25% of children or adults consume the recommended number of servings of fruit and vegetables daily [11,12].

In recent years, children and adults have also decreased their physical activity and increased sedentary behaviors such as watching television, or playing video and computer games [13]. Current guidelines for children suggest 60 min of moderate-intensity exercise on most days of the week [14]. Guidelines for adults include either 150 min of moderate-intensity a week or 75 min of vigorous-intensity exercise a week [15]. Over 50% of children and adults do not meet these recommended levels of daily physical activity [13].

Children learn from the behavior and teaching of their parents, from the experiences provided by parents, and from positive rewards, praise, encouragement, and acknowledgement for their efforts and accomplishments [13,16]. Children's lifestyle, health beliefs and behaviors are also significantly influenced by parental modeling [17]. Although other forces such as school, community, peers, and television become more important as children grow older, the relationships between parents' and children's eating habits, exercise, and body weight continue into adulthood [18].

Parents influence children's eating habits through availability of particular foods, portion size and mealtime structure [19]. Parental modeling also influences children's fruit and vegetable consumption [20-22]. Parents who eat few high fat foods and more fruit and vegetables, and who limit sugared beverages provide their children with invaluable nutrition knowledge that may help them manage overweight and prevent obesity later in life [23]. Also, children whose parents model positive exercise behaviors have been shown to have better exercise self-efficacy and exercise behaviors [24].

Most interventions to treat overweight in children have targeted only the child, with minimal parental input, though parental involvement has been shown to be a central component of most effective interventions [25-28]. Therefore, the intervention described here targets both children and parents, focuses on low-income families in rural areas and includes minorities as well as non-Hispanic whites.

### Theoretical framework for the intervention

Weight stabilization and weight loss require that children and parents learn not only new nutrition and exercise behaviors, but also new coping behaviors. Studies have suggested that when an individual cannot cope effectively with a problem, the individual has less confidence for dealing with the next problem [29]. Social cognitive theory posits that learning and then practicing a new behavior enhances self-efficacy, which in turn increases the probability that the new behavior will be maintained [30-34]. Weight management for children and parents can be difficult to achieve when coupled with frustration and low self-efficacy [35]. However, coping skills training can assist both children and adults in dealing with problems and stress [36,37]. Children and parents who develop skills in communication, goal setting, problem solving, conflict resolution, and positive reinforcement should be more able to make healthy nutrition and exercise behavior decisions and manage their weight. Practicing coping skills should improve health behaviors and eating and exercise self-efficacy. In turn, with improved health behaviors and self-efficacy, children should be able to stabilize their BMI percentile and adiposity trajectory and parents should be able to decrease their BMI and adiposity. Therefore, the intervention developed for this trial provides both nutrition and exercise education and coping skills training, including training in cognitive restructuring, assertiveness training, conflict resolution, and social problem solving skills around nutrition and exercise issues.

## Aims

The first aim of the trial is to examine the effects of a two-phased intervention on weight status, adiposity, nutrition and exercise health behaviors, and self-efficacy in overweight or obese 2^nd^, 3^rd^, and 4^th ^grade children and their overweight or obese parents. The primary outcome in children is stabilization of the BMI percentile trajectory from baseline to 18 months. The primary outcome in parents is a decrease in BMI from baseline to 18 months. Secondary outcomes for both children and parents include improvements in adiposity, nutrition and exercise health behaviors, and self-efficacy from baseline to 18 months. A secondary aim of the trial is to examine in the experimental group the relationships between parent's and children's changes in weight status, adiposity, nutrition and exercise health behaviors, and self-efficacy. Finally, an exploratory aim is to determine whether African American, non-Hispanic white and bilingual Hispanic children and parents in the experimental group benefit differently from the intervention in terms of weight status, adiposity, health behaviors, and self-efficacy.

## Methods

### Design

**Figure 1:**
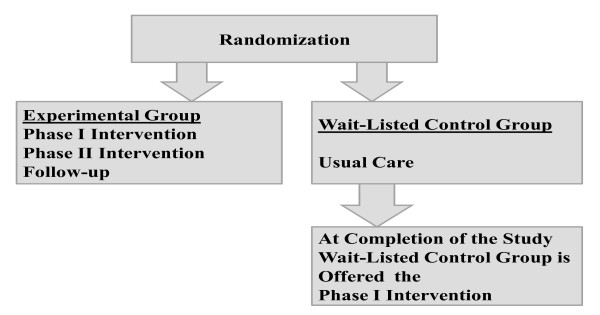
**Randomization for the Trial**.

Family Partners for Health is a 5-year cluster randomized controlled trial with a two-group repeated measures design (See Figure 1). The sample consists of African American (63%), non-Hispanic white (32%), and bilingual Hispanic (5%) low-income, 2^nd ^to 4^th ^grade overweight or obese children and their overweight or obese parents from small towns and rural areas of North Carolina. An experimental group of children and parents are receiving a two-phase intervention with follow-up. In Phase I (Intensive Intervention) they receive 60 minutes of nutrition and exercise education and coping skills training and 45 minutes of exercise training once a week for 12 consecutive weeks. In Phase II (Continued Support) they meet for 9 monthly meetings. They are then followed for 6 months after the completion of Phase II to assess the maintenance of results (follow-up), for a total of 18 months in the trial. The experimental children and parents have data collected at 4 time points: Time 1 (Baseline), Time 2 (12 weeks: Post Phase I-Intensive Intervention), Time 3 (12 months: Post Phase II-Continued Support), and Time 4 (18 months: 6-months of follow-up).

A wait-listed control group of children and parents are receiving usual care, and they have data collected at the same time intervals as the experimental children and parents (Times 1-4). After they complete the Time 4 data collection, they will be offered the nutrition and exercise education, coping skills training, and exercise intervention (Phase I only). During the course of the trial they are receiving monthly cards to thank them for their continued participation in the trial and remind them when they will be eligible to receive the intervention.

### Settings

Eight elementary schools are being used as sites during early evening hours for recruitment and delivery of the intervention. Four schools are located in Wilson, North Carolina, and four in Burlington, North Carolina. The sites are similar in structure, size, and rural, ethnic and racial mix. Each site has classroom space with tables and chairs that are being used for the education and coping skills training classes and a gymnasium that is used for the exercise classes.

There have been eight induction periods over the course of the trial. Forty-four to 45 children and 44-45 parents in two schools (experimental and wait-listed control group) have been enrolled in each period, for a total of 358 children and 358 parents. Each site was randomly assigned to either the experimental or wait-listed control group the first time a cohort was enrolled. The second time that the site had children and parents enrolled, the opposite condition was assigned.

### Power analysis

Power calculations were performed with POWERLIB20 SAS/IML modules [38]. These methods calculate power for the general linear multivariate model, which includes repeated measures data structures, of which a two-group longitudinal design is a special case. Power was based on a separate multivariate model for each outcome addressed in the aims, incorporating measurements from all time points. Models were derived separately for children and parents. The power analysis focused on one representative variable in each group of variables, BMI percentiles (children) and BMI (parents) for weight status, waist circumference (children and parents) for adiposity, the Child and Adolescent Trial for Cardiovascular Health (CATCH) nutrition knowledge subscale (children) and the Health Promoting Lifestyle Profile II nutrition knowledge subscale (parents) for health behaviors, and the Child and Adolescent Trial for Cardiovascular Health (CATCH) self-efficacy subscale (children) and the Eating Self-Efficacy Scale subscale (parents) for self-efficacy. On the basis of pilot data, the autoregressive correlation parameter was chosen as 0.99 for parents' BMI, 0.90 for children's BMI and parents' and children's waist circumference, and 0.84 for the health behavior and self-efficacy variables. Effect sizes used were based on studies with similar designs using the dependent variables of interest. Given the clustered nature of the design (with each site-by-condition classification considered a cluster), an intracluster correlation coefficient of 0.01 was selected (based on BMI). A total of 179 child-parent pairs per group or a total of 358 children and 358 parents have been inducted.

### Sample

The trial was approved by the Institutional Review Board at The University of North Carolina at Chapel Hill. Two months before each induction period, the project manager contacted the principals in the two schools to set up a time for study staff to do classroom presentations and hand out backpack flyers to the children containing information about the benefits of participating in the trial. The students took the flyers home and shared them with parents. Interested parents filled out the flyers with their name, telephone number, and a convenient time for study staff to call. The flyers were then returned to school and put in a box in each teacher's room. The study staff picked up the flyers twice a week and then conducted an initial screening over the telephone. Inclusion criteria for children were ability to speak, write, and read in English; a BMI ≥ 85th percentile for age and gender; a parent or guardian with a BMI ≥ 25 kg/m^2^; and residence with that parent or guardian. The parents were asked their child's gender, age, height, and weight over the telephone, and a BMI percentile was calculated. Inclusion criteria for parents or guardians included an ability to speak, write, and read in English; a BMI ≥ 25 kg/m^2 ^and residence with a 2nd, 3rd or 4th grade child with a BMI ≥ 85th percentile for age and gender. Parents were asked their height and weight over the telephone, and a BMI was calculated. If the child and parent met the trial criteria, an appointment was made that was convenient for them to meet at the school after school hours to confirm eligibility and review the consent and assent. At times, more than one parent or child from the same family wanted to join the trial. If this occurred, we explained that both parents and/or another 2^nd^, 3^rd^, or 4^th ^grade child could participate; however, the same parent and same child had to consistently complete the data forms. Parents could choose among themselves. If there were two children from the same family, the child chosen to fill out the data forms was picked by a coin toss. The project manager confirmed eligibility, explained the trial, and answered all questions before asking the parent to consent and the child to assent. Next a nurse conducted a basic sports history and physical examination on both the child and parent to ensure that they did not have any conditions that would exclude them from participating in the trial. They were excluded if either had a history of a heart murmur, congenital heart disease, family history of sudden death or psychological problems such as claustrophobia that would prevent participation in group classes, or if they were participating in another weight management or prevention program or trial.

### Pilot study

A randomized controlled pilot study was conducted to test the feasibility of the child and parent components of the intervention [39,40]. The intervention was designed to deliver nutrition and exercise information that was applicable across all cultures and sensitive to individual participants' needs, using examples and handouts depicting all three ethnicities and both genders. The nutrition education classes, which focused on making better food choices and controlling portions, and included ethnic menu plans that reduced fat and calories; exercise education focused on increasing activity and decreasing sedentary behaviors. Exercise physiologists taught the exercise education classes and the exercise training classes in the gymnasium, which included basketball, dancing, tag, exercise bicycles, rowing machines, and stair climbing. Parents also attended coping skills training classes.

From the pilot study, several important lessons were learned that informed the main trial [39,40]. First, parents felt it was important for their children to receive the coping skills training. Second, children and parents felt more hands-on activities and food models would make the classes more interesting. Third, parents felt coming to the exercise class twice a week was difficult with their schedules and suggested getting information on how to build a home exercise program. Using this information from the pilot, we revised the intervention for the main study.

### Main trial: phase I (intensive intervention)

The nutrition and exercise education in Classes 1-5 were designed to teach children and parents to work together to develop healthy eating habits and increase exercise. Classes 6-11 were designed to teach children and parents to practice new coping skills, using cognitive restructuring, social problem solving, assertiveness training, and conflict resolution in regard to nutrition and exercise issues [37,41-43]. The exercise training classes were designed to reinforce the importance of increasing activity and decreasing sedentary behaviors. At all classes a light healthy meal and beverages were available. Childcare and homework help were available for other children who came with parents and the children enrolled in the study. Transportation vouchers were available for parents to assist with gas to get to and from the intervention.

Class 1 focused on understanding calories, proteins, carbohydrates, and fats [44,45]. The interventionists used an interactive strategy with pictures in a game format to show participants how calories added up, what foods contained healthy and unhealthy calories, and what foods contained proteins, carbohydrates (simple and complex), and fats (saturated and unsaturated). Foods that were culturally preferred were used. Class 2 taught how portion control could make a difference and what a usual portion size should be [44,45]. The interventionists used food models that children and parents could handle so they could learn to estimate normal portion sizes. Children and parents used measuring cups, bowls, and plates to create a portion-controlled meal balanced in protein, carbohydrates and fats. Each child and parent received a set of measuring cups to take home. Class 3 focused on how to make healthy substitutes with food [44,45]. The interventionists used a card game with pictures of healthy foods that could be substituted for unhealthy foods, including skim or 1% milk for whole milk; lean ground turkey for ground beef; baked or broiled chicken or fish for fried chicken or fish; egg whites for regular eggs; and unsweetened cereals for sweetened cereals. Class 4 used an interactive game to teach children and parents how to choose healthy foods when eating out [44,45]. They learned that it was important not to eat super-size meals, to avoid breaded and fried meats, and to drink water, low fat milk, or sugar-free drinks instead of regular soda, juice, or milkshakes. They also learned how to ask for nutritional information in fast food restaurants and to check for information online. Children and parents then competed in teams. Each team received a menu with nutritional information from a fast food restaurant and worked together to create a balanced meal. Class 5 taught children and parents the importance of exercise and current exercise guidelines [14,15,46,47]. Children and parents learned what moderate-intensity exercise was and how they could use breathing or heart rate to monitor exercise intensity [48].

The remainder of the class sessions focused on coping skills training. Class 6 taught children and parents how to use cognitive restructuring, using recognition of thoughts and feelings, problem solving, and guided self-dialogue to find ways to increase daily exercise [43]. Class 7 used social problem-solving to improve nutrition behaviors. Children and parents learned the problem solving steps, including identifying the problem, determining goals, generating alternative solutions, examining consequences, choosing the solution, and evaluating outcomes [37]. Class 8 used assertiveness training with role playing to teach children and parents how they could motivate each other in a positive manner and provide each other with positive reinforcement [41]. Class 9 used the social problem solving steps used in Class 7 to help children and parents improve exercise behaviors [37]. Class 10 used assertiveness training to teach children and parents how they could get back on track after relapsing from healthy eating and exercise behaviors [41]. Class 11 focused on conflict resolution: children and parents were taught respectful ways to work through conflicts around nutrition and exercise behaviors [42]. Class 12, the final class, included a jeopardy game with questions from previous classes and provided an opportunity for children and parents to review the important components of the intervention.

### Main trial: exercise training classes

Exercise training classes were held weekly for 45 min after the classroom sessions, providing experiences that did not require sophisticated equipment, so that the children and parents could develop a home exercise program. All sessions started with a warm-up, followed by strength circuit training and aerobics, and activities such as small-group team sports (basketball, soccer, floor hockey, and bucketball), chase games, and cardio kickboxing to a video, walking, and Dance Dance Revolution. The exercise interventionists reinforced ways to increase exercise such as taking a walk with a partner, spouse, and/or child, walking the dog, using the stairs instead of the elevator, parking farther from the store, and walking to the store. Each child and parent received a pedometer to measure steps. They were asked to increase their steps by 500 a day for the first week and then by 500 steps a week until they were averaging 10,000 steps a day [48].

### Main trial: phase II (continued support)

During Phase II, the experimental group came back to the school in the early evening once a month for 9 months for continued support. The sessions focused on discussing and problem solving any nutrition and exercise issues participants were having difficulty with. Two weeks after each monthly meeting, the interventionists made a brief telephone call or sent an email to check in and answer any questions the children or parents had.

### Measurement

Table 1 shows the variables and measures being used in the study, the data source, and measurement times. Data are being collected at Time 1 (Baseline), Time 2 (Post Phase I-Intensive Intervention), Time 3 (Post Phase II-Continued Support), and Time 4 (6-Months After Completion of Phase II). All instruments were evaluated for psychometric properties before the pilot study, were re-evaluated during the pilot study, and will be re-evaluated in this study. Completion of measures takes approximately 60 min for most children and parents.

**Table 1:** Summary of measures

Variables and Their Measurement	Respondent	T1	T2	T3	T4	Alpha	Reference
Weight Status Outcomes							

Height	Child/Parent	X	X	X	X	-----	[49]

Weight	Child/Parent	X	X	X	X	-----	[49]

Body Mass Index Percentiles Calculation	Child	X	X	X	X	-----	[49]

Body Mass Index Calculation	Parent	X	X	X	X	-----	[49]

Adiposity Outcomes							[49]

Waist Circumference	Child/Parent	X	X	X	X	-----	[50]

Triceps and Subscapular Skinfolds	Child/Parent	X	X	X	X	-----	[51,52]

Health Behavior Outcomes							

Health Behavior Survey	Child/Parent	X	X	X	X	0.79-0.86	[53]

Health Promoting Lifestyle Profile II	Parent	X	X	X	X	0.78-0.93	[54]

Self-Efficacy Outcomes							

Child and Adolescent Trial for Cardiovascular Health	Child	X	X	X	X	0.87-0.90	[55]

Eating Self-Efficacy Scale	Parent	X	X	X	X	0.88-0.94	[56]

Exercise Self-Efficacy Scale	Parent	X	X	X	X	0.86-0.92	[33]

Sociodemographic Data	Parent for Self and Child	X				-----	-----

T1 (Baseline); T2 (Post Phase I-Intensive Intervention); T3 (Post Phase II-Continued Support); T4 (6-Month After Completion of Phase II)

To ensure inter-rater reliability during the course of the study, RAs were trained and tested for inter-rater reliability prior to each data collection on height, weight, waist circumference, triceps and subscapular skinfolds. During data collection, a duplicate measures program was implemented for quality control testing. Every 10th participant had height, weight, waist circumference, triceps and subscapular skinfolds repeated by a second RA. These data were analyzed monthly to assess reliability and if reliability was not adequate, the RAs were retrained. All data collection RAs were blinded to the study group assignment.

### Sociodemographic data

Parents filled out a demographic sheet for themselves and their children, on age, gender, ethnicity, and race. Additional questions for parents included marital and employment status, socioeconomic status and education level. Additional questions for parents to answer about their children included their birth order and health status.

### Weight outcomes

Height was measured on all children and parents in street clothes without shoes, using a stadiometer, calibrated in 1/8-cm (cm) intervals. Height was measured twice and averaged. Weight on all children and parents was measured in a private room, in street clothes without shoes, to the nearest 0.1 kg using a Tanita WB-110A Digital Scale.

For children, BMI percentiles were calculated twice by entering height, weight, age, and gender [49]. Children with a BMI ≥ 85^th ^and < 95^th ^percentile for age and gender were considered overweight, and those at or above the 95^th ^percentile were obese [49]. BMI of parents was calculated twice by entering height and weight (kg/m^2^) [49]. In adults age 20 years and older, overweight was defined as a BMI between 25.0 and 29.9, and obesity was defined as a BMI equal to or greater than 30.0 [49].

### Adiposity outcomes

Waist circumference was measured in a privately screened area by two RAs, following the procedure used in the Insulin Resistance Atherosclerosis Study, using a Figure Finder measuring tape with lock (Novel Products Inc., Rockton, IL) [50]. All measurements were performed three times and averaged according to the National Health and Nutrition Examination survey procedures [51,52].

Using Lange skinfold calipers, triceps and subscapular skinfolds were measured in children and parents on the right side of the body three times and averaged, also according to the National Health and Nutrition Examination survey procedures [51,52]. To ensure reliability, prior to each data collection, RAs were tested for inter-rater reliability by calculating correlations when measuring skinfolds on the same participants.

### Health behavior outcomes

The 23-item Adult Health Behavior Survey [53] and the 20-item Child Health Behavior Survey [53] were used to collect information on intake of sugared beverages, juices, fruits, vegetables, whole grains, fried, baked and fast foods on a daily or weekly basis. Responses are scored from 0 = none or never to 5 = 5 or more. Alpha coefficients in children range from 0.79 to 0.80 and in parents from 0.80 to 0.86 [53].

The Health Promoting Lifestyle Profile II (HPLP II) was used to measure health promoting lifestyle behaviors in parents [54]. This 48-item, 4-point Likert scale questionnaire with 4 response choices: never, sometimes, often, or routinely, measures the frequency of health promoting behaviors in six subscales. Only four subscales (health responsibility, exercise, nutrition, and stress management) were used in this study; the communication and spirituality subscales were not used. The instrument has been used with both minority and white populations [54]. Alpha coefficients ranged from 0.78 to 0.93 for the subscales. Test-retest reliabilities in African American women have ranged from 0.70 to 0.74 [57].

The Child and Adolescent Trial for Cardiovascular Health (CATCH) questionnaire was used to measure children's diet and exercise health behaviors and self-efficacy [55]. The instrument contains 130 forced-choice items on a 3-point Likert scale in seven subscales: exercise, dietary knowledge, dietary intentions, dietary choices, support, social reinforcement, and self-efficacy. Alpha coefficients ranged from 0.76 to 0.84 in a sample of 5,000 children [55]. The questionnaire is at a 2rd grade reading level and takes approximately 15 min to complete [40]. Alpha coefficients for the subscales ranged from 0.87 to 0.90 in our pilot study [40].

### Self-efficacy outcomes

The Eating Self-Efficacy Scale [56] was used to measure self-efficacy related to dietary changes in parents. This 25-item instrument asks participants to rate their difficulty in controlling eating from 1 (no difficulty) to 7 (difficulty) on two subscales, negative affect (NA) and socially acceptable circumstances (SAC). Negative affect eating is related to emotional eating and the triggers that cause it (e.g., anger or anxiety). Socially acceptable eating is related to overeating at parties, family events, or holidays. Scores range from 25 to 175, with higher scores indicating more difficulty in controlling eating. Alpha coefficients were 0.94 for the NA subscale and 0.85 for the SAC subscale. Test-retest reliability was 0.70 in a sample of 600 women and men [56].

Exercise self-efficacy in parents was measured using Bandura's Exercise Self-Efficacy Scale with 18 questions on a 100-point scale, ranging in 10-unit intervals from 0 (cannot do at all) through intermediate degrees of assurance such as 50 (moderately certain can do) to 100 (certain can do) [33]. The questions are added up and divided by 18 to calculate a total Exercise Self-Efficacy score [33]. A higher score indicates greater self-efficacy. Alpha coefficients of the total scale ranged from 0.86 to 0.92 in adult men and women [58].

### Data analysis plan

An intent-to-treat analysis will be used in which all participants are analyzed according to their initial randomized assignment, whether they receive the intervention regularly or not, to preserve the balance of covariates due to randomization and to provide a conservative analysis that does not overestimate intervention effectiveness. Random baseline differences will be accounted for with this approach.

#### Aim 1

To determine the effects of the intervention on weight status, adiposity, health behaviors and self-efficacy, general linear mixed models will be used. Separate random coefficients models will be used to test each outcome for longitudinal differences between the experimental and wait-listed control groups. Advantages of the mixed model approach include its ability to incorporate data from visits that are mistimed as well as certain missing data structures, so that participants who miss one or more visits need not be excluded from the analysis. In addition, random coefficient models accommodate both categorical and continuous covariates.

If a characteristic is found to differ between the groups at baseline, it will be included as a covariate to account for this random imbalance. Additionally, recognizing that certain factors could influence our dependent variables, whether or not they are randomly imbalanced in the two groups, we will control for them as covariates in the models. These variables include asthma, diabetes, low-dose steroids, psychiatric medications (e.g., amitriptyline), cardiac medications (e.g., beta blockers), and recent smoking cessation.

#### Aim 2

The relationships between experimental child changes in weight status, adiposity, health behaviors, and self-efficacy and experimental parent changes in weight status, adiposity, health behaviors, and self-efficacy will also be addressed via the general linear mixed model. Each outcome will be analyzed through a separate random coefficient model. The models will be structured in a fashion similar to that described for Aim 1, but with a different set of predictor variables, in order to examine the associations between improvements in parents and their children. For each model, the children's baseline values for the outcome will be included as a covariate.

#### Exploratory aim

To determine whether African American, non-Hispanic white, and bilingual Hispanic children and parents in the experimental group benefit differentially from the intervention, two indicator variables for African American and Hispanic children and adults, respectively, will be created, and non-Hispanic whites will serve as the referent group. The models described above for Aim 1 will be refitted, with these indicators added as main effect terms. Additionally, pairwise interaction terms between the intervention indicator and each of these ethnic indicators will be added to the models to assess the degree to which the impact of the intervention differs among these three ethnicities.

### Sample characteristics

Baseline characteristics of parents and children are summarized in Table 2. The mean age of the parents was 36.7 years and the great majority were female (93%). Over half (63%) of the parents and children were African American, 32% were non-Hispanic white, and 5% bilingual Hispanic. The mean age of the children was 8.6 years and the majority (56%) was also female.

**Table 2:** Demographic Characteristics of Parents and Children

**Parents****(*n *= 358)**	
Age	36.7 (+/- 8.0) years
Gender	
Female	93%
Male	7%
Ethnicity and Race	
African American	63%
Non-Hispanic White	32%
Bilingual Hispanic	5%
Education	
< 6 grade or less	1%
Middle School	8%
High School or GED	35%
Associates Degree	44%
Baccalaureate Degree	8%
Graduate Degree	4%
Children (*n *= 358)	
Age	8.6 (+/- 1.0) years
Gender	
Female	56%
Male	44%
Ethnicity and Race	
African American	63%
Non-Hispanic White	32%
Bilingual Hispanic	5%
Education	
2^nd ^grade	20%
3^rd ^grade	41%
4^th ^grade	39%
Birth Order	
1^st ^born	43%
2^nd ^born	34%
3^rd ^born	15%
4^th ^born	6%
5^th ^born	2%

The parents' mean BMI was 37.6, which is classified as obese (Table 3). African American parents mean BMI (38.4) was higher than either non-Hispanic white parents' (36.3) or Hispanic parents' BMIs (32.8). The children's mean BMI percentile was 95.2%, which is also classified as obese. African American children (95.5%) and Hispanic children (95.2%) were similar but non-Hispanic white children (94.0%) were lower.

**Table 3:** Weight and adiposity by total sample and by ethnicity of parents and children

Variable	Mean (+/-SD)		Mean (+/-SD)
**Total Parents (*n *= 358)**		**Total Children (*n *= 358)**	
Height	163.9 (+/- 6.6) cm	Height	138.8 (+/- 8.5) cm
Weight	101.2 (+/- 24.2) kg	Weight	49.1 (+/- 14.1) kg
Body Mass Index	37.6 (+/- 8.4)	Body Mass Percentile	95.2 (+/- 6.4)
Waist Circumference	109.4 (+/- 18.4) cm	Waist Circumference	77.5 (+/- 13.6) cm
Subscapular Skinfolds	37.0 (+/- 9.9) mm	Subscapular Skinfolds	22.4 (+/- 10.4) mm
Triceps Skinfolds	37.3 (+/- 10.4) mm	Triceps Skinfolds	24.7 (+/- 8.9) mm
African American Parents		African American Children	
Body Mass Index	38.4 (+/- 8.4)	Body Mass Index Percentile	95.5 (+/- 6.4)
Waist Circumference	111.2 (+/- 17.9) cm	Waist Circumference	78.0 (+/- 13.7) cm
Subscapular Skinfolds	38.6 (+/- 9.9) mm	Subscapular Skinfolds	22.9 (+/- 10.5) mm
Triceps Skinfolds	39.4 (+/- 10.4) mm	Triceps Skinfolds	25.4 (+/- 9.5) mm
Non-Hispanic White Parents		Non-Hispanic White Children	
Body Mass Index	36.3 (+/- 8.2)	Body Mass Index Percentile	94.0 (+/- 8.1)
Waist Circumference	106.5 (+/- 19.0) cm	Waist Circumference	76.4 (+/- 13.5) cm
Subscapular Skinfolds	34.6 (+/- 9.5) mm	Subscapular Skinfolds	21.7 (+/- 10.5) mm
Triceps Skinfolds	36.3 (+/- 8.2) mm	Triceps Skinfolds	23.5 (+/- 8.0) mm
Hispanic Parents		Hispanic Children	
Body Mass Index	32.8 (+/- 5.2)	Body Mass Percentile	95.2 (+/- 5.2)
Waist Circumference	99.6 (+/- 12.1) cm	Waist Circumference	76.6 (+/- 11.0) cm
Subscapular Skinfolds	30.6 (+/- 7.5) mm	Subscapular Skinfolds	19.1 (+/- 6.0) mm
Triceps Skinfolds	30.2 (+/- 9.6) mm	Triceps Skinfolds	23.3 (+/- 6.7) mm

Parents' mean waist circumference was 109.4 cm, which is classified as obese. African American parents (111.2 cm) had higher waist circumferences than non-Hispanic white (106.5 cm) and Hispanic parents (99.6 cm). Parents' mean subscapular skinfolds were 37.0 mm and triceps skinfolds were 37.3 mm, which are both classified as obese. Again, African American parents had higher subscapular (38.6 mm) and triceps skinfolds (39.4 mm) than non-Hispanic white (34.6 mm; 36.3 mm) and Hispanic parents (30.6 mm; 30.2 mm). The children's mean waist circumference was 77.5 cm, which is classified as obese. African American children (78.0 cm) had higher waist circumferences than non-Hispanic white (76.4 cm) and Hispanic children (76.6 cm). The children's subscapular skinfolds were 22.4 mm, and triceps skinfolds were 24.7 mm, both classified as obese. Again, African American children had higher subscapular skinfolds (22.9 mm) and triceps skinfolds (25.4 mm) than non-Hispanic white children (21.7 mm; 23.5 mm) and Hispanic children (19.1 mm; 23.3 mm).

Health behavior outcomes were measured in children using the Child and Adolescent Trial for Cardiovascular Health (CATCH) [55] questionnaire and the Child Health Behavior Survey [53]. On the CATCH mean scores for dietary intention (1.5), usual food choices (1.5), dietary knowledge (1.5), dietary habits (1.4), and support for physical activity (1.4) were moderate. In addition, children reported support for making healthy food choices from parents (1.4), teachers (1.5) and friends (1.6) (See Table 4).

**Table 4:** CATCH health behavior questionnaire

Variable	Mean (SD)
Dietary Intention	1.5 (+/- 0.13)
Usual Food Choices	1.5 (+/- 0.13)
Dietary Knowledge	1.5 (+/- 0.11)
Dietary Habits	1.4 (+/- 0.16)
Support for	1.4 (+/- 0.15)
Physical Activity	
Food Choices(Parent Support)	1.4 (+/- 0.26)
Food Choices(Teacher Support)	1.5 (+/- 0.31)
Food Choices(Friend Support)	1.6 (+/- 0.31)
Diet Self-Efficacy	2.3 (+/- 0.47)
Physical Activity Self-Efficacy	2.5 (+/- 0.45)

The Child Health Behavior Survey [53] measures usual daily and weekly food and beverage intake and found that 52% of the children drank 2 or more sweetened beverages a day, 21% drank 3 or more glasses of milk a day and 45% drank water when thirsty. Eleven percent ate 4 or more vegetable servings a day and 20% had 4 or more servings of fruit a day. When having a snack 46% chose candy, chips, cereals, cookies or cake and 44% chose fruit, vegetables, yogurt or ice cream for a snack (Table 5).

**Table 5:** Child health behavior survey

Variable	Category	Percent
Sweetened Beverages	None	11.7%
	< 1 glass	11.5%
	1 glass	19.5%
	2 glasses	22.6%
	3 glasses	15.6%
	4 or more glasses	13.7%
	Don't Know	5.3%
Milk	None	17.0%
	< 1 glass	10.6%
	1 glass	27.9%
	2 glasses	22.4%
	3 glasses	8.4%
	4 or more glasses	12.3%
	Don't Know	1.4%
Usually Drink	Unsweetened	5.9%
	Juice	26.6%
	Milk	6.2%
	Sweetened drink	9.2%
	Sports drink	5.6%
	Water	44.8%
	Don't know	1.7%
Vegetables	None	12.3%
	< 1 serving	5.0%
	1 serving	28.6%
	2 servings	23.3%
	3 servings	15.9%
	4 or more serving	11.2%
	Don't Know	3.6%
Fruit	None	7.3%
	< 1 serving	5.6%
	1 serving	22.4%
	2 servings	25.1%
	3 servings	16.2%
	4 or more serving	20.4%
	Don't Know	3.1%
Snacks	Candy	6.2%
	Chips	18.9%
	Cereal	7.8%
	Cookies or Cake	13.4%
	Fruit	30.5%
	Vegetables	4.2%
	Yogurt or Ice Cream	8.9%
	Other Snack	10.0%

Health behavior outcomes were measured in parents using the Adult Health Behavior Survey [53] and The Health Promoting Lifestyle Profile II [54]. The Adult Health Behavior Survey [53] measures daily and weekly food and beverage intake. Over 50% of the parents drank 2 or more sweetened beverages a day, 4% drank 3 or more glasses of milk a day and 46% drank water when thirsty. Six percent ate 4 or more servings of vegetables a day and 3% ate 4 or more servings of fruit a day. When having a snack 61% chose candy, chips, cereal, cookies or cake and 22% chose fruit, vegetables, yogurt or ice cream (Table 6).

**Table 6:** Adult health behavior survey

Variable	Category	Percent
Sweetened Beverages	None	18.7%
	< 1 glass	13.9%
	1 glass	15.4%
	2 glasses	21.8%
	3 glasses	14.8%
	4 or more glasses	13.7%
	Don't Know	1.7%
Milk	None	35.8%
	< 1 glass	20.9%
	1 glass	25.7%
	2 glasses	11.5%
	3 glasses	3.6%
	4 or more glasses	0.6%
	Don't Know	1.9%
Usual Drink	Unsweetened	11.5%
	Juice	6.7%
	Milk	0.6%
	Sweetened Drink	29.9%
	Sports Drink	3.9%
	Water	46.1%
	Don't Know	1.4%
Vegetables	None	1.1%
	< 1 serving	7.5%
	1 serving	22.4%
	2 servings	42.5%
	3 servings	19.6%
	4 or more serving	6.2%
	Don't Know	0.8%
Fruit	None	9.5%
	< 1 serving	17.9%
	1 serving	30.5%
	2 servings	25.7%
	3 servings	11.7%
	4 or more serving	2.5%
	Don't know	2.2%
Snacks	Candy	3.9%
	Chips	28.2%
	Cereal	12.9%
	Cookies or Cake	15.9%
	Fruit	8.4%
	Vegetables	2.8%
	Yogurt or Ice Cream	11.2%
	Other Snack	13.1%

The Health Promoting Lifestyle Profile II examines health responsibility, nutrition, exercise, and stress (Table 7). On the subscales the parents mean score for health responsibility (1.3) was sometimes, nutrition (2.2) was often, exercise (0.9) was never to sometimes and stress (1.3) was sometimes.

**Table 7:** Health promoting lifestyle profile II, eating self-efficacy and exercise self-efficacy

	Variable	Total Mean (SD)
HPLPII	Health Responsibility	1.3 (+/- 0.56)
	Nutrition	2.2 (+/- 0.49)
	Exercise	0.9 (+/- 0.56)
	Stress	1.3 (+/- 0.50)

Exercise	Self-Efficacy	45.0 (+/- 21.9)

Eating	Self-Efficacy^1^	
	Negative Affect	43.6 (+/- 21.5)
	Socially Acceptable	37.5 (+/- 13.1)
	Total Scale	80.8 (+/- 31.1)

Eating	Self Efficacy^2^	
	Most difficulty controlling eating	0.22 (+/- 0.49)

Eating Self Efficacy Scale^1^: rescaling 6 to 6.5 for the cohorts received 1 to 6 scale instrument

Eating Self Efficacy Scale^2^: proportion of most difficulty controlling eating (6 & 7)

Self-efficacy in children was measured using the CATCH [55] questionnaire, which examines diet self-efficacy (2.3) and exercise self-efficacy (2.5) (Table 4). Self-efficacy in parents was measured using the Eating Self-Efficacy Scale [56] and the Exercise Self-Efficacy Scale [33]. See Table 7. The children had moderate mean scores on diet self-efficacy (2.3) and physical activity self-efficacy (2.5). The parents demonstrated moderate scores in eating self-efficacy for the negative affect scale (43.6) and socially acceptable scale (37.5). In exercise self-efficacy the parents scored moderately (45.0) on the scale.

## Discussion

Overweight and obesity in lower income ethnic minority adults and children have reached epidemic proportions in the U.S., with no improvements in sight [3,59]. The direct and indirect costs of obesity have increased over the last decade to $300 billion a year [8]. Genetic predisposition coupled with excessive caloric intake, decreased exercise, and increased sedentary behavior contribute to the current epidemic [59].

The data reported here reflect the high overweight and obesity among ethnic minority adults and children. The mean BMI of these parents (37.6 kg/m^2^) was in the obese classification II, which includes a BMI from 35.0 to < 40.0 kg/m^2 ^[49]. African American parents and children had the highest BMIs and BMI percentiles. This is consistent with data from the Centers for Disease Control and Prevention indicating that 79% of African American women are either overweight or obese [1]. The data on the children followed closely the data on their parents, with a mean body mass index percentile of 95%. These children are already obese. This finding is consistent with research indicating that adult overweight and obesity influence child overweight and obesity [59-61]. Adiposity measurements were also very high in this population. The waist circumferences, subscapular skinfolds, and triceps skinfolds of children and parents all indicated of overweight and obesity. African American children had the highest waist circumferences, subscapular skin folds and triceps skinfolds of any group.

Child and parent health behaviors showed generally unhealthy nutritional patterns. The majority of children (52%) and parents (50%) drank two or more glasses of sweetened beverages per day. Only 21% of children and 4% of parents drank three or more glasses of milk per day, though intake of at least three glasses of skim milk a day has been found to help stabilize weight and build strong bones [11,12]. When thirsty, 45% of children chose water and 41% chose either a sweetened beverage, juice, or a sports drink to quench their thirst. Similarly, when thirsty, 46% of adults chose water and 41% chose either a sweetened beverage, juice, or sports drink. Only 11% of children and 6% of adults ate more than four servings of vegetables per day. Of interest, 20% of the children and only 3% of the adults ate four or more servings of fruit per day. The current recommendations are to eat six to eight servings of vegetables and fruit per day [45]. When asked what they ate for snacks, the responses were fairly similar for both children and adults. Children chose chips (19%), cereal (8%), cookies or cake (13%), fruit (31%), or yogurt or ice cream (9%). Adults chose chips (28%), cereal (13%), cookies or cake (16%), fruit (8%), or yogurt or ice cream (11%).

On the Health Promoting Lifestyle Profile II [54] questionnaire, the health responsibility subscale looks at preventive health actions taken and comfort in asking health care providers questions about health. The nutrition subscale examines whether respondents are meeting guidelines for fruit, vegetable, and milk intake. The exercise subscale examines what they do for exercise, how many days a week, and at what intensity. The stress subscale looks at the efforts parents make to minimize stress in their lives. These parents' responses were between never (0) and sometimes (1) in relation to health responsibility, nutrition, exercise, and stress. That is, parents only sometimes or never felt comfortable seeking preventive health care. They sometimes or never ate the recommended servings of six to eight fruits and vegetables per day [45]. Parents only sometimes or never drank the recommended three glasses of skim milk per day [45]. In addition, parents only sometimes or never partook in the recommended number of exercise sessions per week [48].

Parents reported moderate difficulty in controlling eating on both the negative affect and socially acceptable subscales of the Eating Self-Efficacy Scale [56]. Parents also said they had moderate difficulty believing they could exercise in the different situations presented on the Exercise Self-Efficacy Scale [33]. The children had moderate difficulty believing they could make changes to their diet and exercise behaviors.

These data do not reflect a representative sample of African American, non-Hispanic white and bilingual Hispanic children. Further, the data were self report except for weight and adiposity measurements, and bias is always possible with self-report data collection. Despite these limitations, this study provides useful information on sociodemographics, weight, adiposity, health behaviors, and self-efficacy in a large group of children and parents from rural North Carolina. The findings highlight the importance of early intervention, with children and parents partnering together to manage their weight.

## Competing interests

The authors declare that they have no competing interests.

## Authors' contributions

DB is the principal investigator of the study. RM, TS, and AS are co-investigators of the study and contributed to developing the research questions and study design. MS is the day-to-day project manager. DB, RM, TS, AS, MS, MN, and GH contributed equally contributed to implementation of the study protocol. All authors contributed in the development, read and approved the final manuscript.

## Pre-publication history

The pre-publication history for this paper can be accessed here:

http://www.biomedcentral.com/1471-2458/12/250/prepub

## References

[B1] Centers for Disease Control and Prevention Estimated county-level prevalence of diabetes and obesity--United States, 2007 MMWR Morb Mortal Wkly Rep 2009 58 45 1259 1263 19940830

[B2] Yates EA MacPherson AK Kuk JL Secular trends in the diagnosis and treatment of obesity among US adults in the primary care setting Obesity 2011 in press [epub ahead of print] 10.1038/oby.2011.271 21869761

[B3] Ogden CL Carroll MD Curtin LR Lamb MM Flegal KM Prevalence of high body mass index in US children and adolescents, 2007-2008 JAMA 2010 303 3 242 249 10.1001/jama.2009.2012 20071470

[B4] Orsi CM Hall DE Lynch JL Pediatric obesity epidemiology Curr Opin Endocrinol Diabetes Obes 2011 18 1 14 22 10.1097/MED.0b013e3283423de1 21157323

[B5] American Diabetes Association Standards of medical care in diabetes Diabetes Care 2011 34 S11 S61 21193625 10.2337/dc11-S011 PMC3006050

[B6] American Heart Association Heart Disease and Stroke Statistics, 2011 Update 2011 123 Dallas: American Heart Association 10.1161/CIR.0b013e3182009701 PMC4418670 21160056

[B7] Finkelstein EA Trogdon JG Cohen JW Dietz W Annual medical spending attributable to obesity: payer-and service-specific estimates Health Aff 2009 28 822 831 10.1377/hlthaff.28.5.w822 19635784

[B8] Behan DF Cox SH Lin Y Pai J Pedersen HW Yi M Obesity and its relation to mortality and morbidity costs Soc Actuaries 2010 1 61

[B9] Daniels SR The consequences of childhood overweight and obesity Future Child 2006 16 1 47 67 10.1353/foc.2006.0004 16532658

[B10] Newby PK Are dietary intakes and eating behaviors related to childhood obesity? A comprehensive review of the evidence J Law Med Ethics 2007 35 35 60 10.1111/j.1748-720X.2007.00112.x 17341216

[B11] Piernas C Popkin BM Increased portion sizes from energy-dense foods affect total energy intake at eating occasions in US children and adolescents: patterns and trends by age, group, and sociodemographic characteristics, 1977-2006 Am J Clin Nutr 2011 in press [Epub ahead of print] 10.3945/ajcn.110.008466 PMC3192477 21918222

[B12] Poti JM Popkin BM Trends in energy intake among US children by eating location and food source, 1977-2006 J Am Diet Assoc 2011 111 8 1156 1164 10.1016/j.jada.2011.05.007 21802561 PMC3148484

[B13] Yackobovitch-Gavan DM Nagelberg N Phillip M Ashkenazi-Hoffnung L Hershkovitz E Shalitin S The influence of diet and/or exercise and parental compliance on health-related quality of life in obese children Nutr Res 2009 29 6 397 404 10.1016/j.nutres.2009.05.007 19628106

[B14] U.S. Department of Health and Human Services Physical Activity Guidelines for Americans: Children 2008 Washington, D.C.: U.S Department of Health and Human Services

[B15] U.S. Department of Health and Human Services Physical Activity Guidelines for Americans: Adults 2008 Washington, D.C.: U.S. Department of Health and Human Services

[B16] Hoghughi M The importance of parenting in child health: doctors as well as the government should do more to support parents BMJ 1998 316 7144 1545 1550 10.1136/bmj.316.7144.1545 9596585 PMC1113192

[B17] Norton DE Froelicher ES Waters CM Carrieri-Kohlman V Parental influence on models of primary prevention of cardiovascular disease in children Eur J Cardiovasc Nurs 2003 2 311 322 10.1016/S1474-5151(03)00072-0 14667487

[B18] Hoghughi M Long N Handbook of Parenting: Theory and Research for Practice 2004 1 London: Sage

[B19] Patrick H Nicklas TA A review of family and social determinants of children's eating patterns and diet quality J Am Coll Nutr 2005 24 2 83 92 15798074 10.1080/07315724.2005.10719448

[B20] Bere E Klepp KI Correlates of fruit and vegetable intake among Norwegian schoolchildren: parental and self-reports Public Health Nutr 2004 7 8 991 998 15548337 10.1079/PHN2004619

[B21] Galloway AT Fiorito L Lee Y Birch LL Parental pressure, dietary patterns, and weight status among girls who are "picky eaters" J Am Diet Assoc 2005 105 4 541 548 10.1016/j.jada.2005.01.029 15800554 PMC2530930

[B22] Wardle J Carnell S Cooke L Parental control over feeding and children's fruit and vegetable intake: how are they related? J Am Diet Assoc 2005 105 2 227 232 10.1016/j.jada.2004.11.006 15668680

[B23] Richie LD Welk GJ Styne D Gerstein DE Crawford PB Family environment and pediatric overweight: what is a parent to do? J Am Diet Assoc 2005 105 5 Suppl 1 S70 S79 15867900 10.1016/j.jada.2005.02.017

[B24] Trost SG Sallis JF Pate RR Freedson PS Taylor WC Dowda M Evaluating a model of parental influence on youth physical activity Am J Prev Med 2003 25 277 282 10.1016/S0749-3797(03)00217-4 14580627

[B25] Rowland K Wallace R Which factors increase the risk of an infant becoming an overweight child? J Fam Pract 2009 58 383 384 19607779

[B26] Berry D Sheehan R Heschel R Knafl K Grey M Family-based interventions for childhood obesity: a review J Fam Nurs 2004 10 429 449 10.1177/1074840704269848

[B27] Oude Luttikhuis H Baur L Jansen H Shrewsburty VA O'Malley C Stork RP Interventions for treating child obesity: a cochrane review Cochrane Rev 2009 14 4 1571 1729 10.1002/14651858.CD001872.pub2 19160202

[B28] Bautista-Castano I Doreste J Serra-Majem L Effectiveness of interventions in the prevention of childhood obesity Eur J Epidemiol 2004 19 7 617 622 15461192 10.1023/b:ejep.0000036890.72029.7c

[B29] Marlott GA Gordon JR Relapse Prevention: Maintenance Strategies in Addictive Behavior Change 1985 New York: The Guilford Press

[B30] Bandura A Social Learning Theory 1977 Englewood Cliffs: Prentice-Hall

[B31] Bandura A Self-efficacy mechanism is human agency Amer Psychol 1982 37 122 147

[B32] Bandura A Social Foundations of Thought and Action: A Social Cognitive Theory 1986 Englewood Cliffs: Prentice Hall

[B33] Bandura A Self-efficacy: The Exercise of Control 1997 New York: W.H. Freeman

[B34] Bandura A Health promotion by social cognitive means Health Educ Behav 2004 31 12 143 164 15090118 10.1177/1090198104263660

[B35] Thompson JK Coovert MD Richards KJ Johnson S Cattarin J Development of body image, eating disturbance, and general psychological functioning in female adolescents: covariance structure modeling and longitudinal investigations Int J Eat Disord 1995 18 3 221 236 10.1002/1098-108X(199511)18:3<221::AID-EAT2260180304>3.0.CO;2-D 8556018

[B36] Forman SG Linney JA Brondino MJ Effects of coping skills training on adolescents at risk for substance abuse Psychol Addic Behav 1990 4 67 76

[B37] Forman SG Coping Skills Training for Children and Adolescents 1993 San Francisco: Jossey-Bass

[B38] Muller KE LaVange LM Ramey SL Ramey CG Power calculations for general linear multivariate models including repeated measures applications J Am Stat Assoc 1992 87 1209 1226 10.2307/2290663 PMC4002049 24790282

[B39] Berry D Grey M Melkus G Savoye M Preliminary testing of an intervention for multiethnic overweight and obese parents of at risk for overweight and overweight children Obes Res 2005 13 S1 A88 10.1016/j.apnr.2006.01.007 PMC1945054 17481469

[B40] Berry D Savoye M Melkus G Grey M An intervention for multiethnic overweight and obese parents and overweight children Appl Nurs Res 2007 20 63 71 10.1016/j.apnr.2006.01.007 17481469 PMC1945054

[B41] Davidson M Boland EA Grey M Teaching teens to cope: coping skills training for adolescents with diabetes mellitus J Soc Pediatr Nurs 1997 2 65 72 10.1111/j.1744-6155.1997.tb00062.x 9152897

[B42] Deutsch M Brickman E Conflict resolution Pediatr Rev 1994 15 1 16 22 10.1542/pir.15-1-16 8121841

[B43] Beck AT Cognitive Therapy and the Emotional Disorders 1979 New York: Penguin Press

[B44] Gidding SS Dennison BA Birch LL Daniels SR Gilman MW Lichtenstein AH Rattay KT Steinberger J Stettler N Van Horn L Dietary recommendations for children and adolescents: a guide for practitioners Pediatrics 2006 117 2 544 559 16452380 10.1542/peds.2005-2374

[B45] U.S. Department of Health and Human Services Dietary Guidelines for Americans 2005: Chapter 3 Weight Management 2005 Washington, DC: U.S. Department Health and Human Services

[B46] Deforche B Haerens L de Bourdeaudhuij L How to make overweigh children exercise and follow the recommendations Inter J Pediatr Obes 2011 6 S1 35 41 10.3109/17477166.2011.583660 21905814

[B47] Andersen RE Wadden TA Validation of a cycle ergometry equation for predicting steady-rate VO2 in obese women Med Sci Sports Exerc 1995 27 10 1457 1460 8531619

[B48] American College of Sports Medicine Guidelines for Exercise Testing and Prescription 2006 7 New York: Lippincott Williams and Wilkins

[B49] Centers for Disease Control and Prevention What is BMI? 2004 http://www.cdc.gov/nccdphp/dnpa/bmi/bmi-adult.htm

[B50] Haffner SM Howard G Mayer E Insulin sensitivity and acute insulin response in African-Americans, Non-Hispanic whites, and Hispanics with NIDDM: the Insulin Resistance Atherosclerosis Study Diabetes 1997 46 63 69 10.2337/diabetes.46.1.63 8971083

[B51] National, Heart, Lung and Blood Institute Clinical Guidelines on the Identification, Evaluation, and Treatment of Overweight and Obesity in Adults: The Evidence Report 1998 Washington, DC: National Institutes of Health 1 262

[B52] National, Heart, Lung, and Blood Institute The Practical Guide to Identification, Evaluation, and Treatment of Overweight and Obesity in Adults 2000 Washington, DC: National Institutes of Health 1 94

[B53] North Carolina Department of Health and Human Services Adult and Child Healthy Behavior Surveys: Physical Activity and Nutrition Behavior Monitoring Form 2004 Raleigh: Women and Children's Health Section

[B54] Walker SN Sechrist KR Pender NJ The health-promoting lifestyle profile: development and psychometric characteristics Nurs Res 1987 36 76 81 3644262

[B55] Parcel GS Edmundson E Perry CL Feldman HA O'Hara-Tompkins N Nader PR Johnson CC Stone EJ Measurement of self-efficacy for diet-related behaviors among elementary school children J Sch Health 1995 65 23 27 10.1111/j.1746-1561.1995.tb03335.x 7731197

[B56] Glynn SM Ruderman J The development and validation of an eating self-efficacy scale Cogn Ther Res 1986 10 403 420 10.1007/BF01173294

[B57] Jefferson V Melkus G Spollett G Health promotion practices of young black women at risk for type 2 diabetes mellitus Diabetes Educ 2000 26 295 302 10.1177/014572170002600210 10865595

[B58] Shin YH Jang HJ Pender NJ Psychometric evaluation of the exercise self-efficacy scale among Korean adults with chronic diseases Res Nurs Health 2001 24 24 68 76 11260587 10.1002/1098-240x(200102)24:1<68::aid-nur1008>3.0.co;2-c

[B59] Wang Y Global secular trends in childhood obesity Int J Pediatr Obes in press

[B60] Nowicka P Flodmark CE Family in pediatric obesity management: a literature review Int J Pediatr Obes 2008 3 Supplement 1 44 50 18278632 10.1080/17477160801896994

[B61] Ko J Childhood obesity and familial environmental factor according to the developmental stages: the Korea NHANES study J Korean Acad Fam Med 2008 29 939 947

